# A Front-end Redesign With Implementation of a Novel “Intake” System to Improve Patient Flow in a Pediatric Emergency Department

**DOI:** 10.1097/pq9.0000000000000263

**Published:** 2020-02-27

**Authors:** Kevin P. Carney, Ann Crespin, Gray Woerly, Nicholas Brethouwer, Jeff Baucum, Michael C. DiStefano

**Affiliations:** From the * Department of Pediatrics, Section of Emergency Medicine, University of Colorado School of Medicine, Children’s Hospital Colorado, Aurora, Colo.; † Emergency Department, Children’s Hospital Colorado, Aurora, Colo.

## Abstract

**Introduction:**

Children’s Hospital Colorado is an academic, tertiary-care Level 1 Trauma Center with an emergency department (ED) that treats >70,000 patients/year. Patient volumes continue to increase, leading to worsening wait times and left-without-being-seen (LWBS) rates. In 2015, the ED’s median door-to-provider time was 49 minutes [interquartile range (IQR) = 26–90], with a 3.2% LWBS rate. ED leadership, staff, and providers aimed to improve patient flow with specific goals to (1) decrease door-to-provider times to a median of <30 minutes and (2) decrease annual LWBS rate to <1%.

**Methods:**

An inter-professional team utilized quality improvement and Lean methodology to study, redesign, and implement significant changes to ED front-end processes. Key process elements included (1) new Flow Nurse/EMT roles, (2) elimination of traditional registration and triage processes, (3) immediate “quick registration” and nurse assessment upon walk-in, (4) direct-bedding of patients, and (5) a novel “Intake” system staffed by a pediatric emergency medicine physician.

**Results:**

In the 12 months following full implementation of the new front-end system, the median door-to-provider time decreased 49% to 25 minutes (IQR = 13–50), and the LWBS rate decreased from 3.2% to 1.4% (a 56% relative decrease). Additionally, the percentage of patients seen within 30 minutes of arrival increased, overall ED length-of-stay decreased, patient satisfaction improved, and no worsening of the unexpected 72-hour return rate occurred.

**Conclusions:**

Using quality improvement and Lean methodology, an inter-professional team decreased door-to-provider times and LWBS rates in a large pediatric ED by redesigning its front-end processes and implementing a novel pediatric emergency medicine-led Intake system.

## INTRODUCTION

Patient crowding is a problem facing emergency departments (ED) worldwide.^1–3^ Causes of crowding include increased use of EDs, patient boarding in the ED, increased patient complexity, and inefficient ED operations.^4^ Crowding leads to longer wait times to see providers, patient safety concerns, worse outcomes in certain clinical scenarios, and decreased patient satisfaction.^2,5,6^ There is an increased national focus on this important health topic, with the Center for Medicare and Medicaid services identifying multiple operational metrics as key to evaluating the quality of care provided in an ED.^7^ The American Academy of Pediatrics also recognizes this as a particular problem affecting the care of pediatric patients in the ED and in 2015 published a report outlining best practices for patient flow and care for these patients.^8^

A key driver of ED patient flow is its “front-end system,” consisting of all the operational steps that occur before a provider sees the patient. Strategies employed to improve the front-end processes include the abolishment of traditional nurse-led triage, “split-flow” models that create separate patient streams depending on each individual’s particular care needs, direct-bedding of patients, and placing providers in triage.^8–11^ A Physician in Triage and other models utilizing non-physician providers can decrease door-to-provider times and decrease left-without-being-seen (LWBS) rates.^12–18^ Most reports of patient flow improvements come from general EDs, where the majority of patients are adults; thus, there are few reports of how similar strategies may impact pediatric-focused EDs.^19,20^

**Fig. 1.**
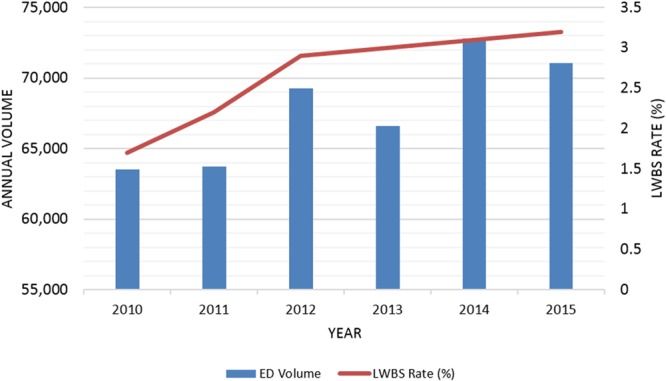
Annual ED volumes and LWBS rates.

Children’s Hospital Colorado has seen increased patient volumes and LWBS rates since moving into a new hospital in 2008 (Fig. 1). In 2016, the ED leaders, staff, and providers wanted to improve patient flow via a large-scale front-end system redesign. The purpose of this report is to share the change process, specific operational changes implemented, and the resulting impact on patient flow in this tertiary-care pediatric ED.

### Specific Aims

The specific SMART aims were to redesign the front-end system by January 2017 with a goal to (1) decrease median door-to-provider times from 49 minutes to <30 minutes and (2) decrease annual LWBS rate from 3.2% to <1% by the following year.

## METHODS

This study was approved by the institution’s Organizational Research Risk and Quality Improvement Panel (ORRQIRP). The ORRQIRP was established by agreement between the academic institution’s human subject research review board and the study institution in 2011. ORRQIRP is sanctioned by the institutional review board to review quality improvement (QI) project proposals to determine if they do not meet the criteria for human subjects research.

### Setting

This project took place in the ED of a 395-bed tertiary care, academic freestanding children’s hospital. The hospital is a Level 1 Trauma Center with a 48-bed ED that sees over 70,000 patients/year and has a 13% admission rate. ED medical providers include Pediatric Emergency Medicine (PEM) physicians, general Pediatricians, Advance Practice Providers (APPs), PEM fellows, residents (Pediatric, Emergency Medicine, and Family Medicine), and medical students.

### Improvement Methods

The ED leadership team met in early 2015 to discuss improving operational flow. With the support of hospital executive leadership, the ED hired a process improvement specialist to help with these efforts. The ED Medical Director and Assistant Clinical Nurse Manager formed an ED Operations Committee in June 2015 to help lead the initial PDSA cycles and educate staff.

**Fig. 2.**
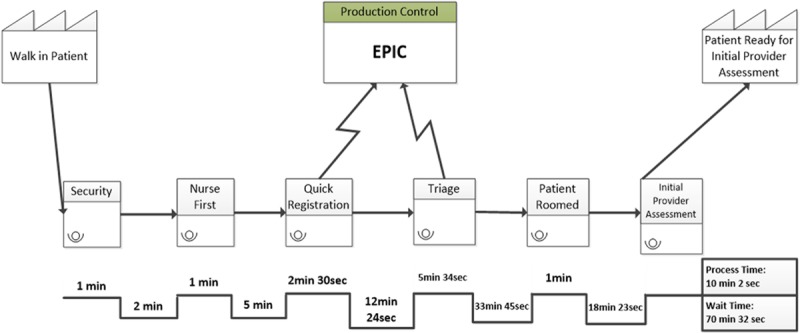
Value-stream MAP of original front-end system.

Starting in June 2016, an expanded inter-professional team including >20 members of ED leadership, physicians, APPs, nurses, EMTs, and registration staff members began meeting to plan further large-scale improvement efforts. The team employed QI and Lean methods to study the current system, including process-mapping of the front-end system and subsequent development of a value-stream map. The team determined that of the average 80-minutes patients spent waiting to see a provider, only 10 minutes was spent in-process, of which <3 minutes was considered value-added to the patient (Fig. 2). With a goal of operational changes in place by January 2017, the team decided to hold a 5-day Kaizen^21^ event to expedite system implementation.

The team met in November 2016 for the Kaizen and spent the first 3 days using Lean methodology^22^ to remove redundant and non-value-added steps from the front-end system. Steps removed included questions previously placed in the triage process by other QI efforts but not considered critical to the front-end process. Important questions such as patient/family safety questions and learning preferences were moved to later portions of the visit. The team developed new front-end processes (described below) and piloted the new system for 8 hours on Kaizen Day 4. The team observed the process during this initial trial and made changes both in real-time and at the Day 5 session. Concurrent with the clinical process development, the team engaged with Information Technology, Compliance, Facilities, and other hospital services to change crucial components of the Electronic Health Record (EHR) and waiting room physical layout to accommodate the new process.

After the 5-day Kaizen, the team wanted to test the new front-end system once more before official implementation. The team chose the following Monday (historically the highest volume day of the week) to test the system for 18 hours. The team arrived early and provided “just-in-time” training for the staff and providers. Despite seeing over 260 patients that day (making it 1 of the 10 highest-volume days of 2016), the team observed no significant safety or operational issues. The following day, the team resolved some small outstanding issues, and the new front-end system “went live” the next day on November 16, 2016—9 days after the start of the Kaizen. Volunteer “system super users” from the Kaizen team and ED clinical leaders provided 2 weeks of 24 hours/day on-the-ground support. Project leaders sent staff weekly updates with key metrics for 2 months after implementation.

## INTERVENTIONS

### Staff and Provider Education

To prepare staff for the anticipated operational changes, the Operations Committee began educational efforts in the summer of 2015. Didactics, open forums, and staff “town halls” allowed for staff to learn the basic theories of ED operations, patient flow, and the importance of front-end processes.

### “Flow Nurse and Flow EMT” Roles

In the summer of 2015, the Operations Committee also worked to develop “Flow Nurse” and “Flow EMT” roles. These departmental roles have no direct patient assignments, but rather are responsible for overall department flow. Tasks include rooming patients from the waiting room, greeting ambulance arrivals, and facilitating room turnover. These roles are staffed every day from 11 am to 1 am to coincide with maximum patient volumes and were implemented in December 2015.

### Implementation of Parallel “Quick” Registration and “Sorter Nurse” Processes

**Fig. 3.**
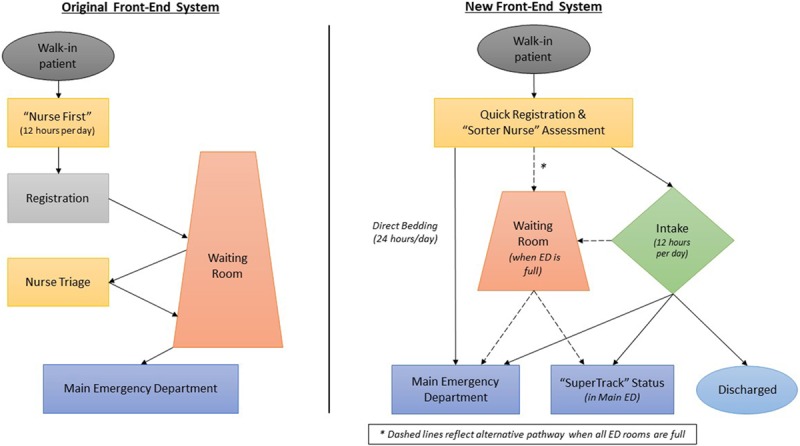
Patient flow diagram of original vs new front-end system.

To expedite registration and clinical assessment of walk-in patients, the Kaizen team discontinued the original linear steps of patient registration and nurse-led triage processes. Instead, the team developed a parallel process that occurs immediately after patient arrival consisting of “quick registration” and initial nurse evaluation (Fig. 3). The process:

• Walk-in patients are greeted immediately upon arrival by a Patient Access team member and “Sorter Nurse”
• Patient Access team member performs “Quick Registration” while the nurse assesses the patient
• “Quick Registration” consists of:
  - Documenting the patient’s name, date-of-birth, gender
  - Obtaining a patient digital photo for the EHR
  - Documenting who brought the patient to the ED (eg, parent, grandparent, etc.)
  - Caregiver signs “consent-to-treat” form
  - Placing identification wristband on the patient
• “Sorter Nurse” assesses the patient and:
  - Identifies critical illness requiring immediate rooming
  - Documents chief complaint; any other key details
  - Assesses patient using Pediatric Assessment Triangle^23^
  - Assigns Emergency Services Index (ESI) acuity level
  - Records weight
  - Records medication allergies
  - “Sorts” patient to either “Emergent Bed,” “Direct Bed” or “Intake” status
    ▪ Emergent bed criteria *(patient brought immediately to a room and registered in the ED room):*
      • All ESI Level 1s
      • Immunocompromised patients
      • Concern for high-risk infectious pathogen (eg, fever in a returning traveler)
    ▪ Direct Bed criteria:
      • All ESI Level 2s
      • Patients referred from another healthcare facility
      • Concern for TB, varicella, measles, or pertussis
      • Families registering >2 patients
      • Patient/caregiver speaks a language other than English or Spanish
      • Social concerns
      • Mental Health chief complaints
    ▪ Intake criteria:
      • All other patients not meeting “Emergent” or “Direct Bed” criteria when Intake system is open (see below)

### Implementation of “Direct Bedding”

The previous front-end system required multiple linear steps before placing the patient in an ED room (Fig. 3). “Direct Bedding” means patients are immediately roomed after registration and a brief nursing assessment. In the new system, this process occurs 24 hours/day when beds are available. After rooming, the bedside nurse completes and documents a “Secondary Assessment,” consisting of:

• Focused history and exam
• Vital signs
• Past medical history
• Current medications
• Pain level
• Note: the Bedside Nurse may change the Sorter Nurse’s ESI triage based on further information

### Development of “Intake” System

The Kaizen team developed, piloted, and implemented a new “Intake” system, which is open daily from 11 am to 11 pm. Intake operates in the 4 previously used triage rooms. The Intake team includes a PEM physician, scribe, nurse, and EMT who work to assess patients rapidly, determine a disposition, and initiate orders (when appropriate). The process:

• Sorter Nurse determines if the patient is appropriate for Intake (see above criteria)
• EMT rooms patient and obtains vital signs
• PEM physician evaluates patient while a scribe documents in the EHR
• PEM physician places orders for medications, labs, or radiology studies (if needed)
• PEM physician determines patient disposition:
  - Discharge from ED
  - Roomed in ED
    ▪ “SuperTrack”—a patient expected to discharge home within 1 hour of Intake evaluation. These patients usually require a simple clinical reevaluation or laboratory/radiology test.
    ▪ “Main ED”—a patient expected to require >1 hour of further history-taking, work-up, consultations, or treatment.
• If a patient is discharged from Intake, the Intake RN discharges the patient and escorts them to the registration check-out desk
• If roomed, the ED “Flow RN” monitors EHR for notification of “SuperTrack” or “Main ED” disposition and escorts the patient from Intake to room.

### Measures

Primary outcome measures consisted of door-to-provider times and LWBS rates. “Provider” is defined as a resident, fellow, pediatrician, PEM attending, or APP.

Secondary outcome measures included the percentage of patients seen <30 minutes after arrival, overall length-of-stay (LOS), and patient satisfaction as measured by standardized hospital-wide post-visit surveys (PRC, Omaha, Neb.). The unanticipated patient returns to the ED within 72 hours (% of total visits) were tracked as a balancing measure.

### Data Analysis

The team extracted operational data from the hospital’s EHR, Epic Systems Corporation (Verona, Wisc.), and summarized the continuous outcomes of door-to-provider times and LOS with medians and interquartile ranges (IQR). Groups were compared using Wilcoxon rank-sum tests. χ^2^ tests were utilized to compare LWBS percentage, percent of patients seen <30 minutes, and 72-hour return rates. The team created Statistical Process Control charts using Minitab Statistical Software (Minitab LLC, State College, Pa.).

## RESULTS

**Fig. 4.**
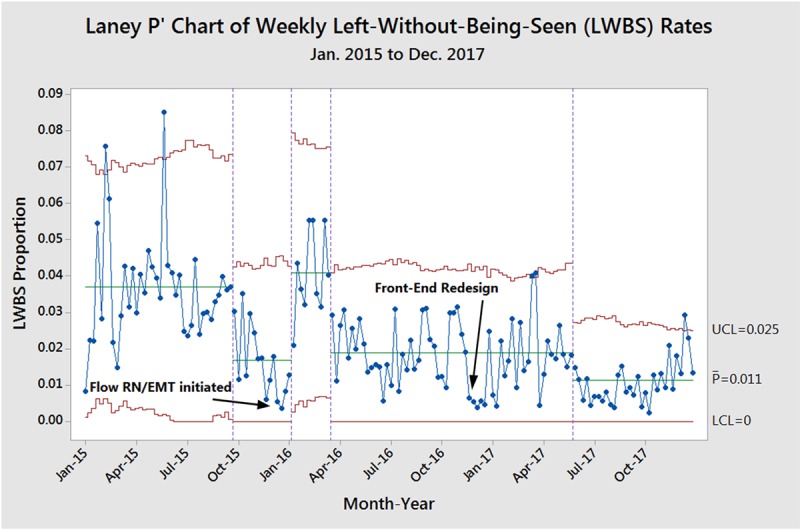
Laney P’ chart of LWBS rates rate by week.

We compared the 12-month post-implementation operational metrics to baseline operational data from 2015 (Table 1). In 2015, the ED had 70,088 patient visits compared with 73,394 visits in the 12-month post-implementation period (a 5% increase). For the primary outcome measures, the post-implementation median door-to-provider time improved to 25 minutes (IQR 13–50), a nearly 50% decrease compared with the 2015 baseline of 49 minutes (IQR 26–90). In addition, the LWBS rate decreased from 3.2% in 2015 to 1.4% in the 12 months post-implementation. An annotated Laney P’ chart demonstrates LWBS rates and shows an overall decrease in weekly variation in the system compared with the 2015 baseline (Fig. 4).

**Table 1.**
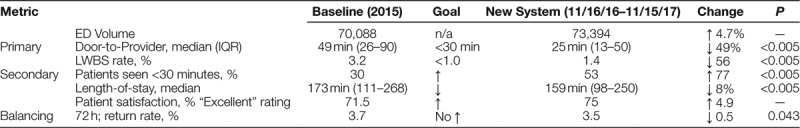
ED Operational Metrics: Baseline Versus 1 Year Post-Implementation

**Fig. 5.**
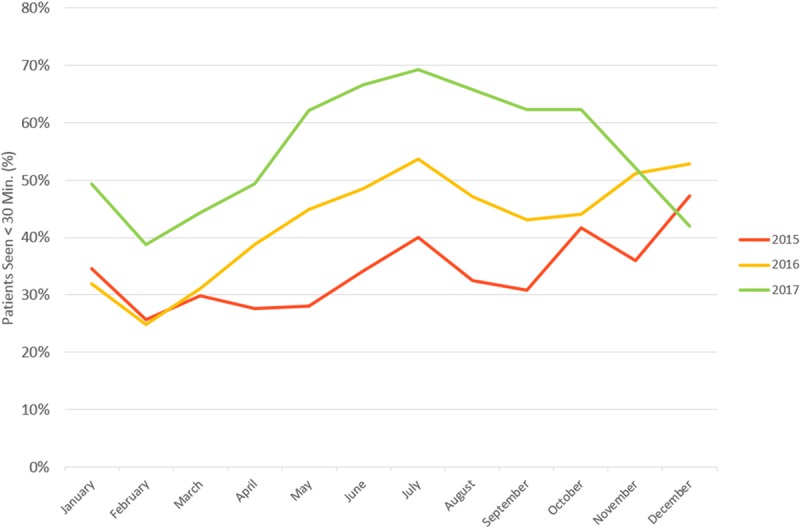
Monthly percentage of patients seen <30 minutes by year (2015–2017)

All secondary outcome measures improved in the 12 months post-implementation. The percentage of patients seen <30 minutes rose to 53%, a 77% relative increase compared with the 2015 baseline. Figure 5 shows the increasing monthly percentage of patients seen in <30 minutes year-over-year between 2015 and 2017. The median LOS decreased from 173 to 159 minutes (8% decrease), including improvement for both admitted patients (4.5% decrease) and discharged patients (9% decrease). Overall, patient satisfaction increased from 71% who reported the visit as “excellent” in 2015 to 75% in 2017. Finally, the 72-hour return rates did not worsen following the implementation of the new system (Table 1).

## DISCUSSION

Much of the literature on ED operations has focused on general EDs, where adults make up the majority of patients; thus, it is not fully known how previously described front-end principles apply to pediatric EDs.^20^ To the authors’ knowledge, this project is the first to describe the implementation of a front-end system in a pediatric ED utilizing a split-flow model, including direct-bedding and a PEM-staffed Intake system. This project shows that by utilizing Lean methodology, QI principles, and knowledge of ED operational principles, a large pediatric ED can realize similar patient flow improvements to those seen in adult systems. As hoped, the new front-end system drove patient flow by decreasing door-to-provider times, thereby improving LOS for all patients, and improved functional ED capacity allowing for a decreased LWBS rate. Of note, patient flow metrics improved despite a 5% increase in volume compared with the baseline period.

One could argue the observed operational improvements are a result of increased staffing rather than process redesign. The original front-end system was inefficient as it included many non-value-added processes leading to frequent patient flow bottlenecks due to queueing theory. Merely adding staff to this inefficient system would not have made a meaningful improvement in patient flow. By decreasing the number of front-end steps (namely the discontinuation of nurse-led triage and the implementing direct-bedding), the new system allows for a decreased door-to-provider time by removing non-value-added steps rather than increased staff. The newly developed PEM-led Intake system is an adjunct to this more efficient system and allows for earlier initiation of care and saves critical ED bed space by facilitating rapid discharge of patients who need no further care.

Direct-bedding is a strategy frequently found in other front-end redesigns to help reduce door-to-provider times.^9,24^ It is a critical component in the success of our new front-end system but has challenges when the ED is full, and direct-bedding is no longer an option. At these times, we must enact backup processes to bring a nurse or EMT from the main ED to the waiting room to obtain vital signs and initiate standing orders. Also, in certain situations, patients previously assigned to a direct bed status may be seen in Intake by the PEM physician when there is no ED capacity. These backup processes ensure patient care continues despite the lack of room availability.

Patients and families report wait times as a key driver of satisfaction with pediatric ED visits.^25^ As expected, with our nearly 50% decrease in door-to-provider times, parent visit satisfaction increased from 71% to 75%. While a notable improvement, opportunities exist for further an improved experience as the ED continues to experience large swings in patient volumes over the day and throughout the year. Despite improvement efforts, we continue to experience periods when wait times become excessive, and patients decide to leave-without-being-seen. Of note, outliers on the LWBS statistical process control chart (Fig. 4) largely coincide with weeks of high patient volume and resulting increased door-to-provider times. Expectantly, patient visit satisfaction decreases during these periods.

Previous studies attempted to calculate the financial impact of crowding and the return-on-investment of various front-end redesigns.^26–29^ Due to the hiring of a process improvement specialist, and modest increases in staffing, the estimated incremental cost of our new system is $400,000/year. While a formal financial analysis is yet-to-be performed, the operational improvements are expected to yield positive financial gains. In 2015 nearly 2,300 patients left without a provider evaluation compared with approximately 1,000 in the 12 months following the front-end redesign. The difference of 1,300 is the number of patients who would have been expected to walk out in the previous system but now are seen and incur visit charges. If the ED maintains improved patient flow performance, we expect to realize a positive financial return while also providing a better patient care experience for patients and families.

### Limitations

This project has several limitations to consider. First, as a project performed at a single pediatric ED, results may not be translatable to other institutions that have different local barriers to patient throughput. Second, we obtained financial support from hospital executive leadership to hire a Process Improvement specialist as well as make modest staff increases, investments other institutions may not be in a position to make. Finally, given our large number of ED providers and staff, we were able to utilize volunteers to have a “system superuser” in the department 24 hours each day for 2 weeks after implementation of the new system. This type of support may not be possible in smaller EDs with more limited staff.

### Next Steps

The next steps include future PDSA cycles to improve backup plans for when the ED is full and direct bedding is not possible. This intervention includes an analysis of the sorting process to ensure safe and accurate assessments to minimize the risk of patients clinically decompensating while in the waiting room. Criteria for which patients are appropriate for the Intake system will be evaluated and adjusted as necessary to maintain adequate patient flow in the Intake system. Further analysis of the sub-processes in each patient stream (direct-bedding and Intake) will help identify opportunities to improve efficiency and decrease system variation. Finally, a formal financial analysis is planned to determine the impact of the system.

## CONCLUSIONS

Using QI and Lean methodology, an inter-professional team in a large, tertiary-care pediatric ED designed and implemented a novel front-end system and significantly improved patient flow by decreasing door-to-provider times 49% and LWBS rates by over 50%. Key concepts included decreasing non-value-added steps in the front-end and implementing a split-flow system utilizing direct-bedding and a PEM-led Intake system to drive patient flow. The system has led to a meaningful improvement of overall ED LOS for all patients and improvement of patient satisfaction scores. Future work will focus on maintaining these improvements during high-volume times of the day and throughout the year.

## DISCLOSURE

The authors have no financial interest to declare in relation to the content of this article.

## ACKNOWLEGEMENTS

The authors would like to thank the staff and providers of the Children’s Hospital Colorado Emergency Department for their unwavering commitment to improving the care they provide their patients and the CHCO executive leaders for support of the project.
